# Small Island Effects on the Thermal Biology of the Endemic Mediterranean Lizard *Podarcis gaigeae*

**DOI:** 10.3390/ani13182965

**Published:** 2023-09-19

**Authors:** Aikaterini Reppa, Ariadne Faidra Agori, Panayiota Santikou, Aristeidis Parmakelis, Panayiotis Pafilis, Efstratios D. Valakos, Kostas Sagonas

**Affiliations:** 1 Section of Ecology and Taxonomy, Department of Biology, National and Kapodistrian University of Athens, 157 72 Athens, Greece; 2 Section of Animal and Human Physiology, Department of Biology, National and Kapodistrian University of Athens, 157 72 Athens, Greece; 3 Section of Zoology and Marine Biology, Department of Biology, National and Kapodistrian University of Athens, 157 72 Athens, Greece; ppafil@biol.uoa.gr (P.P.); evalakos@biol.uoa.gr (E.D.V.); 4 Zoological Museum, National and Kapodistrian University of Athens, 157 72 Athens, Greece; 5 Section of Zoology, Department of Biology, Aristotle University of Thessaloniki, 541 24 Thessaloniki, Greece

**Keywords:** ectotherms, Greece, islands, lacertidae, podarcis, skyros, thermal adaptations

## Abstract

**Simple Summary:**

As ectotherms that do not produce metabolic heat to regulate their body temperature, lizards largely rely on the thermal quality of the environment for most aspects of their biology. To compensate for geographically induced changes in the thermal environment, different lizard populations within a single species should either regulate their behavior or shift their thermal preferences, responding to directional selection. Here, we studied the thermal ecology of the endemic Skyros wall lizard (*Podarcis gaigeae*) and assessed the influence of thermal habitat quality on body temperature and preferred body temperatures. Our findings suggest that the species thermoregulate effectively in a wide variety of habitats and support the view that the thermal characteristics of certain species are plastic and respond to environmental changes.

**Abstract:**

Ectotherms are vastly affected by climatic conditions as they rely on external sources of heat to regulate their body temperature, and changes in their habitat thermal quality could seriously affect their overall biology. To overcome the problems of a thermally unfavorable habitat, lizards need to either adjust their thermoregulatory behavior or respond to directional selection and shift their preferred body temperatures. To assess the impact of habitat thermal quality on the thermoregulatory profile, we studied multiple islet and ‘mainland’ populations of the Skyros wall lizard *Podarcis gaigeae*, an endemic lacertid to Skyros Archipelago, Greece. We evaluated the effectiveness of thermoregulation (*E*) using the three main thermal parameters: body (*T*_b_), operative (*T*_e_), and preferred (*T*_pref_) temperatures. We first hypothesized that the spatial homogeneity, the scarcity of thermal shelters, and the exposure to higher winds on islets would result in more demanding climate conditions. Second, we anticipated that islet lizards would achieve higher E in response to the lower thermal quality therein. As hypothesized, thermal parameters differed between populations but not in the expected manner. Skyros ‘mainland’ habitats reached higher temperatures, had more intense fluctuations, and were of lower thermal quality. As a result, lizards showed higher accuracy, precision, and effectiveness of thermoregulation. Noteworthy, we found that lizards from different populations have shifted their thermal profile and preferred body temperatures to cope with the particular conditions prevailing in their habitats. The latter supports the labile view on the evolution of thermoregulation.

## 1. Introduction

Island life is known to promote rapid evolution and diversification in animals [1,2]. A combination of factors, including relaxed predation pressure, food scarcity, and increased intraspecific competition on islands [3], has been proposed to explain shifts in body size [2], feeding apparatus, digestive performance [4], locomotion [5], and head morphology [6]. Likewise, recent studies have shown that the poorer spatial heterogeneity and milder thermal environments of islands compared to mainland habitats [3,7,8] are important drivers of biogeographical variations in reptilian body temperature and thermoregulation patterns as well [9]. Lizards, as ectotherms that cannot produce metabolic heat to control their body temperature, largely depend on the temperature of their environment and habitat use [10]. In other words, a lizard’s thermal habitat governs the whole’s fitness, performance, and survival [11]. Investigating the response of saurian thermal physiology to directional selection could, therefore, be the missing piece of the puzzle for a better understanding of lizards’ adaptive and evolutionary potential in a changing environment.

Compared to other animals, lizards face disproportionally the challenges associated with rising temperature [12,13], including loss of thermally suitable (micro-)habitat and geographic range shifts that alter species interactions [14]. These changes often result in population declines and threaten the survival of numerous lizard species [15,16]. To overcome the problems associated with shifts in ambient environmental temperatures, lizards have to either select different habitats or undergo changes in their thermal preferences [17]. Lizard populations and/or species with the ability to adapt their optimal body temperature to achieve thermal equilibrium with the environment have better chances to survive [18]. 

To depict the thermal profile of ectotherms, three parameters are traditionally used [19]. First, body temperatures (*T*_b_) refer to the temperature animals achieve in the field. Second, the preferred body temperatures (*T*_pref_) that animals achieve in the lab under no ecological restrictions, and third, the operative temperatures (*T*_e_) that a non-thermoregulating animal reaches under natural conditions. Taken together, these metrics can provide information on an animal’s precision, accuracy, and effectiveness of thermoregulation [19]. In unsuitable thermal environments, lizards are expected to display active thermoregulation to avoid thermal stress. In less demanding environments, on the other hand, lizards tend to shift their thermoregulatory strategy to a certain extent of thermoconformity [19].

To assess whether lizard thermoregulation responds to directional selection, we focused on the endemic Skyros wall lizard (*Podarcis gaigeae*; Skyros Archipelago, Greece) and estimated the precision, accuracy, and effectiveness of thermoregulation in various populations. During the sea level rise in the Aegean Sea in the last 18,000 years, a number of islets emerged at Skyros Archipelagos, which *P. gaigeae* colonized, evolving a number of physiological and life-history traits, e.g., [20,21,22]. A previous study on the thermal biology of the species showed that the increase in body size in islet populations could partially explain the less effective thermoregulation of islet lizards. Here, we questioned whether the isolation and the different conditions prevailing on the islets could drive differences in the thermoregulatory profile of the species. To address this, we worked with five islet and five “mainland” Skyros populations in order to detect presumptive differences in the thermoregulatory profile due to distinct habitat features. As the contribution of habitat diversity is more intense at finer scales [23], the thermal quality of small islands and/or islets may be influenced by habitat diversity more than that of larger islands. Hence, environmental homogeneity on islets can become a main determinant of a lizard’s thermoregulatory strategy. Using multiple populations of a single species, we minimized phylogenetic signal effects [24] on thermoregulation and provided the necessary power to detect habitat effects on lizard thermal strategy. Although Skyros populations are not separate populations in the strictest sense, see, e.g., [25], they dwell in different habitat types.

Climate conditions on islands are more benign compared to the mainland, with less temperature fluctuations and extreme values due to the buffering sea effect [3,7]. However, the scarcity of thermal shelters, the very small area, the low altitude, and exposure to high winds that occur on islets often eliminate the mild conditions characterizing larger islands [26,27]. We thus hypothesized that the islets would have lower and less favorable thermal quality compared to the “mainland” sites (i.e., Skyros Island). Second, we presumed that these harsher thermal conditions would influence the effectiveness of thermoregulation, and islet lizards would exhibit higher *E* values (i.e., active thermoregulation) than their “mainland” Skyros conspecifics [26]. Finally, we investigated whether the different populations of *P. gaigeae* that are exposed to different climatic conditions adapted their thermal biology accordingly; see also [28].

## 2. Materials and Methods

### 2.1. Study System

The Skyros Archipelago (38°51′ N, 24°33′ E) (Central Aegean Sea, Greece) comprises the main island of Skyros and 21 surrounding islets of varying sizes, the majority of which harbor the endemic Skyros wall lizard (*P. gaigeae*). *Podarcis gaigeae* is an insectivorous, small-bodied (snout-to-vent length—SVL: 40–73 mm), lacertid lizard that lives in most biotopes (e.g., pine forests, semi-dunes, phrygana, and maqui vegetation, rocky sites) [29]. The islet populations got isolated due to rising sea levels and have been separated from the main island (hereafter “mainland”) from 1500 to 9500 years ago [30].

**Figure 1:**
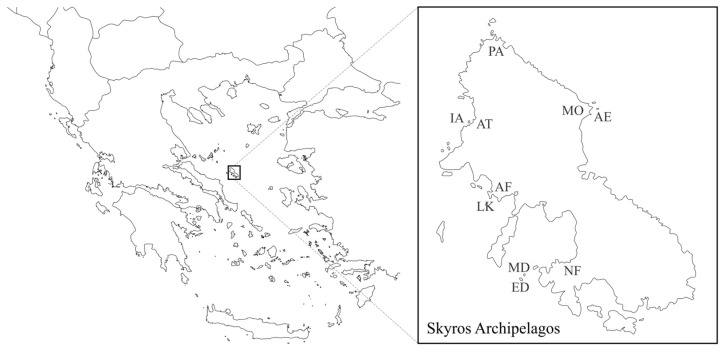

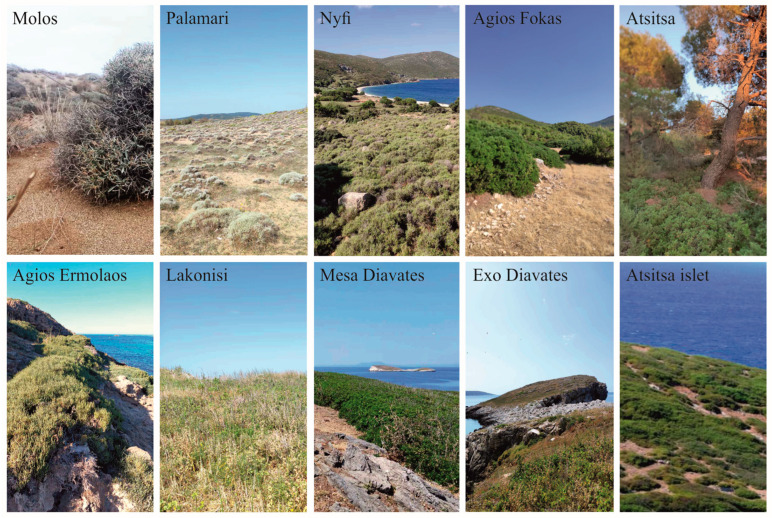
Map of Greece focusing on the Skyros Island and its surroundings islets. The letter codes denote sampling localities. PA to Palamari; IA to Islet of Atsitsa; AT to Atsitsa; MO to Molos; AE to Agios Ermolaos; AF to Agios Fokas; LK to Lakonisi; NF to Nyfi; ED to Exo Diavates and MD to Mesa Diavates. The sampling areas differ in vegetation type and cover.

We carried out the fieldwork from mid-May to early June 2022 in five “mainland” (Nyfi, Agios Fokas, Molos, Palamari, and Atsitsa) and five islet (Lakonisi, Mesa and Exo Diavates, Atsitsa Islet, and Agios Ermolaos) populations (Figure 1). The biotope among mainland and/or islet sites differs remarkably in terms of predation regime (Skyros hosts several specialized lizard predators, such as snakes and birds, while islets are predator-free [31]) and vegetation density. Palamari and Molos are semi-dunal biotopes; Nyfi and Agios Fokas vegetation consists of dense phrygana shrubs and maquis, while Atsitsa habitat is a dense pine forest. All islets are rocky, and their vegetation typically consists of sparse phrygana shrubs and, in some cases, nitrophilous plants. Vegetation coverage differs among the five islets: at one extreme (dense vegetation) lies Atsitsa Islet, followed by the moderate-density vegetation islets of Lakonisi, Agios Ermolaos, Mesa Diavates, and Exo Diavates (with the lowest density and coverage) (Figure 1).

### 2.2. Thermal Measurements

#### 2.2.1. Field Temperatures (Body and Operative Temperatures; *T*_b_ and *T*_e_)

We sampled *T*_b_ of 473 adult male and female lizards in equal sex ratios in all populations (see Table 1 for population’s sample size). Lizards were caught by nose by one person who carefully slipped the noose over the head of the lizard and pulled it. A second person (KS) carefully released the lizard by loosening the noose and measured the body temperature to the nearest 0.1 °C with a quick-reading cloacal thermometer (T-4000, Miller & Weber, Inc., Queens, NY, USA). To minimize thermal shifts due to stress or contact with the handler, the temperature of each lizard was measured within 10 s of capture [32,33]. We further recorded the length (snout-vent length; SVL), mass, sex, and condition of lizard immediately after capture. Each lizard was then placed in an individual cotton bag of 25 × 25 cm. All gravid females found to have oviductal eggs (by ventral palpation) were excluded from sampling to avoid any reproductive-derived bias [33]. SVL (in mm) and mass (in g) were measured with Digital Calipers (Silverline 380244, Silverline, London, UK, accurate to 0.01 mm) and a digital scale (i500 Backlit Display, My Weight, London, UK, accurate to 0.1 g), respectively. All lizards were kept in the lab for a period of two months and then were released back at the site of sampling.

We estimated *T*_e_ of non-regulating lizards [34] by using 28 hollow, electroformed copper models connected to seven data loggers (HOBO U12 4-Channel External Data Logger-U12-008) [35]. Copper models were closed at both ends, approximated the size, shape, and color of the focal species, and were filled with 2–3 mL of water in order to simulate lizard’s heat storage capacity [34,36]. To certify the similarity of thermal responses between models and lizards, we tested and compared the cooling and heating rates [36], as has been described in Sagonas et al. [28]. Operative temperatures were recorded for a day from 7:40 to 19:50 at 10 min intervals. Models were placed randomly to cover all main types of microhabitats available to lizards in order to better sketch out the thermal profile of each site [37,38]. We categorized microhabitats based on sunlight exposure (full light [FL], semi-light [SL], shade [S]) and substrate type (litter [L], soil [S], rock [R]).

**Table 1:** Values for snout-vent length (in mm), mass (in gr), body (*T*_b_), preferred (*T*_pref_) and operative (*T*_e_) temperatures are given for the ten *Podarcis gaigeae* populations. We also provide descriptive statistics for the deviation of *Ts*_e_ from *T*_set_ (*d*_e_) and the deviation of *T*_b_ from *T*_set_ (*d*_b_). Means ± standard deviation; range; sample size (N). The indices of effectiveness of thermoregulation. E_H_ refers to the equation of Hertz et al. [19], whereas E_B_ to Blouin-Demers and Weatherhead [39].

	Population	SVL (mm)	Mass (g)	T_b_ (°C)	T_pref_ (°C)	T_e_ (°C)	d_b_ (°C)	d_e_ (°C)	E_H_	E_B_
Skyros Island (“mainland”)	Agios Fokas (AF)	6.30 ± 0.50 (5.40–7.30) *N* = 27	5.52 ± 1.63 (3.31–8.95) *N* = 27	31.39 ± 2.98 (25.40–38.00) *N* = 43	32.56 ± 1.35 (29.07–35.42) *N* = 27	39.11 ± 8.86 (23.90–70.80) *N* = 28	1.17 ± 1.41 (0.0–5.57) *N* = 43	5.87 ± 7.11 (0.0–35.51) *N* = 28	0.80	4.70
	Atsitsa (AT)	4.54 ± 1.67 (2.10–8.10) *N* = 31	5.57 ± 0.99 (2.65–7.10) *N* = 31	30.24 ± 3.72 (21.30–35.90) *N* = 48	31.31 ± 1.74 (28.15–35.13) *N* = 31	34.92 ± 8.35 (19.40–62.90) *N* = 28	1.65 ± 2.21 (0.0–8.47) *N* = 48	5.07 ± 6.40 (0.0–29.77) *N* = 28	0.67	3.42
	Molos (MO)	5.70 ± 0.42 (4.50–6.50) *N* = 25	4.81 ± 1.32 (2.35–7.77) *N* = 25	33.06 ± 2.48 (26.80–39.10) *N* = 60	32.97 ± 0.90 (31.37–34.80) *N* = 25	41.48 ± 8.65 (22.40–73.30) *N* = 28	0.95 ± 1.29 (0.0–5.20) *N* = 60	8.00 ± 7.59 (0.0–38.83) *N* = 28	0.88	7.05
	Nyfi (NF)	6.19 ± 0.53 (5.30–7.10) *N* = 23	5.98 ± 1.64 (3.93–9.36) *N* = 23	32.03 ± 3.22 (24.90–37.80) *N* = 52	32.23 ± 1.96 (28.82–35.52) *N* = 23	38.79 ± 8.23 (22.30–66.80) *N* = 28	0.98 ± 1.31 (0.0–5.21) *N* = 52	6.53 ± 6.69 (0.0–32.34) *N* = 28	0.85	5.56
	Palamari (PA)	5.54 ± 0.54 (4.70–6.70) *N* = 18	4.00 ± 1.42 (2.57–7.70) *N* = 18	33.20 ± 2.69 (27.20–37.40) *N* = 57	33.27 ± 1.28 (30.77–35.37) *N* = 18	42.06 ± 9.11 (25.40–72.30) *N* = 28	0.66 ± 1.12 (0.0–4.34) *N* = 57	7.29 ± 8.19 (0.0–36.60) *N* = 28	0.91	6.63
Islet	Agios Emolaos (AE)	5.79 ± 0.64 (4.50–6.80) *N* = 29	4.58 ± 1.58 (2.60–7.49) *N* = 29	32.73 ± 2.57 (28.70–37.70) *N* = 39	33.54 ± 0.85 (31.93–35.40) *N* = 29	40.29 ± 8.83 (26.30–69.50) *N* = 28	1.29 ± 1.38 (0.0–4.03) *N* = 39	6.44 ± 7.55 (0.0–34.14) *N* = 28	0.80	5.16
	Atsitsa Islet (AI)	6.42 ± 0.52 (5.10–7.40) *N* = 28	6.03 ± 1.73 (3.24–9.73) *N* = 28	30.39 ± 1.85 (28.00–35.20) *N* = 38	32.42 ± 0.63 (31.30–33.65) *N* = 28	37.66 ± 9.77 (24.90–71.20) *N* = 28	1.40 ± 1.08 (0.0–3.30) *N* = 38	4.61 ± 9.11 (0.0–36.99) *N* = 28	0.70	3.21
	Exo Diavates (ED)	7.67 ± 0.93 (6.30–8.80) *N* = 28	12.15 ± 5.01 (6.60–19.51) *N* = 28	32.05 ± 2.87 (25.00–37.60) *N* = 49	34.07 ± 0.94 (32.08–35.20) *N* = 28	40.03 ± 8.53 (24.80–72.60) *N* = 28	1.84 ± 2.32 (0.0–8.23) *N* = 49	5.87 ± 7.89 (0.0–37.60) *N* = 28	0.69	4.03
	Lakonisi (LK)	6.93 ± 0.81 (5.50–8.10) *N* = 25	9.07 ± 3.45 (3.55–15.24) *N* = 25	32.12 ± 2.39 (27.00–38.80) *N* = 45	33.85 ± 0.83 (32.08–35.20) *N* = 25	37.84 ± 8.62 (25.20–70.90) *N* = 28	1.54 ± 1.60 (0.0–5.99) *N* = 45	5.29 ± 6.60 (0.0–35.30) *N* = 28	0.71	3.75
	Mesa Diavates (MD)	5.97 ± 0.65 (4.90–7.00) *N* = 26	4.90 ± 1.75 (2.54–8.04) *N* = 26	32.78 ± 2.79 (28.10–38.60) *N* = 42	33.70 ± 0.95 (31.73–35.27) *N* = 26	38.85 ± 10.62 (25.70–66.70) *N* = 28	1.49 ± 1.50 (0.0–4.64) *N* = 42	6.88 ± 8.55 (0.0–31.66) *N* = 28	0.78	5.39

#### 2.2.2. Lab Measurements (Preferred and Set-Point Temperatures; *T*_pref_ and *T*_set_)

Out of 473 lizards caught in the field, 260 lizards (see Table 1 for each population sample size) were transferred to the animal facilities of the Faculty of Biology at the University of Athens and housed individually in vitreous terraria (20 × 25 × 15 cm) under a controlled photoperiod (12 h light: 12 h dark). Lizards had access to water ad libitum and were fed once every other day with mealworms (*Tenebrio molitor*) coated with mineral powder (TerraVit Powder, JBL GmbH & Co. KG, Neuhofen, Germany). For each lizard, we estimated body condition as the residuals of the linear regression of log10-transformed weight against log10-transformed SVL [40]. Prior to any experimental procedure, lizards remained in the terraria for a 2-week period to acclimatize to the new environment.

Set-point ranges (*T*_set_) of each population were estimated as the central 50% distribution of *T*_pref_ values of each individual [19]. Preferred temperatures were measured every hour for a five-hour period with a cloacal Miller–Weber thermometer in a specially design terrarium (100 × 25 × 25 cm). A thermal gradient ranging from 15 to 60 °C was achieved in the terrarium by placing two ice bags at one end and two incandescent heating lamps (100 and 60 W) at the other end [41]. Before *T*_pref_ recording, we allowed lizards to acclimate for an hour.

#### 2.2.3. Effectiveness of Thermoregulation (E)

The effectiveness of thermoregulation, defined as the ability of an animal to maintain its body temperature closer to its preferred (*T*_pref_) rather than operative (*T*_e_) temperatures, was calculated using two indices. First, we used the formula proposed by Hertz et al. [19]: *Ε* = 1 − (${\bar{d}}_{b}$/${\bar{d}}_{e}$), where ${\bar{d}}_{b}$ denotes the accuracy of thermoregulation and is the mean deviation of field *T*_b_ from *T*_set_ and ${\bar{d}}_{e}$ is the mean deviation of field *T*_e_ from *T*_set_. *E* values range from zero, denoting a thermoconformer individual that selects microhabitats randomly, to one that indicates an individual that actively selects an appropriate microhabitat (i.e., thermoregulator).

Nonetheless, Hertz’s index has been criticized for certain innate biases since different ${\bar{d}}_{b}$ and ${\bar{d}}_{e}$ combinations may lead to the same values of *E*; for details, see [39]. In other words, species that face different thermal environments and thermoregulate with different precision and accuracy may still have the same *E* values. We, therefore, used the alternative index (${\bar{d}}_{e}\;$− ${\bar{d}}_{b}$) proposed by Blouin–Demers and Weatherhead [39] that circumvents this problem. In this index of thermoregulation, the magnitude of the difference between ${\bar{d}}_{e}$ and ${\bar{d}}_{b}$ is a measure of how much an animal departs from thermoconformity. Positive values indicate animals actively thermoregulating, zero demonstrate perfect thermoconformity, while negative values describe animals avoiding thermally favorable habitats.

### 2.3. Statistical Analysis

The Kolmogorov–Smirnov and Lilliefors test was applied to assess the normality of the data. Whenever parametric assumptions were not met, nonparametric tests were applied; otherwise, we used parametric tests. Welch *t*-test to control for unequal sample size and variances was first performed to test for sex-related effects on the accuracy of thermoregulation for each population by evaluating the differences in field body (*T*_b_) and preferred body (*T*_pref_) temperatures between sexes. One-way ANOVA was used to assess population and or habitat (mainland vs. islet population) differences in body length (SVL) and field body temperature (*T*_b_). To identify if significant differences exist between pairs of group means, the post-hoc Games Howell test was employed. Generalized linear models (GLM) fitting a Poisson distribution were used to assess the effects of site and habitat on the accuracy of thermoregulation (${\bar{d}}_{b}$) and the quality of the thermal environment (${\bar{d}}_{e}$). To add on, we used permutation multivariate ANOVA with 9999 iterations to test for differences in *T*_e_, *T*_pref_, and *T*_set_ between populations, while GLMs by applying 9999 permutations and Bray-–Curtis dissimilarity with site as fixed factor were used to assess possible effects of SVL on *T*_pref_ and *T*_est_.

The two indices of effectiveness of thermoregulation (*E* and ${\bar{d}}_{e}\;$− ${\bar{d}}_{b}$) were estimated for each population separately. We used a bootstrap resampling method 1000 times to generate the 95% confidence intervals [19] and applied a Bonferroni correction to adjust the confidence intervals for multiple comparisons. All statistical analyses were carried out in R 4.1.2 [42]. 

## 3. Results

### 3.1. Body Size and Field Measurements (T_b_ and T_e_)

Body size differed significantly between populations with lizards from the islets exhibiting in general longer (SVL; *F*_9,250_ = 28.40, *p* < 0.001) and heavier (*F*_9,250_ = 28.79, *p* < 0.001) bodies that their mainland conspecifics (Table 1). In particular, lizards from Exo Diavates, Lakonisi, and Atsitsa Islet were longer than the rest of the populations, while those from Atsitsa had the smallest size. No further differences were observed (Games Howell test). In addition, we found significant sexual body size dimorphism in Lakonisi (*t* = −4.72, *df* = 16.21, *p* < 0.001), Agios Ermolaos (*t* = −3.95, *df* = 26.94, *p* < 0.001), Exo Diavates (*t* = −3.85, *df* = 25.88, *p* < 0.001), and Molos (*t* = −28.1, *df* = 15.30, *p* = 0.047) populations, with males being larger than females. 

**Figure 2:**
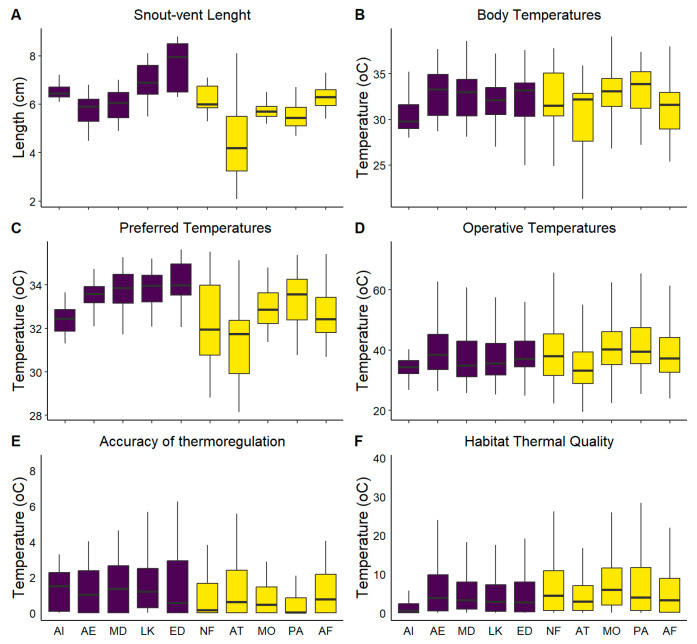
Boxplot of SVL (in cm) and the thermal parameters that define ectotherms’ thermoregulation effectiveness. Purple color indicates islet populations and yellow ‘mainland’ Skyros populations. Snout-vent length (SVL), body temperature (*T*_b_), preferred temperatures (*T*_pref_), operative temperatures (*T*_e_), accuracy of thermoregulation (*d*_b_), and habitat thermal quality (*d*_e_). Lizards on islets were generally longer than their mainland conspecifics (**A**). We also found significant differences in body (**B**), preferred (**C**) and operative (**D**) temperatures between populations, that reflect the differences in vegetation type and cover. Finally, the comparison of the accuracy of thermoregulation (**E**) and the quality of the thermal environment (**F**) showed that mainland lizards had higher accuracy and inhabit more favorable environments.

We found no significant differences between male and female body temperatures for all studied populations (all *P*s > 0.05) despite the observed differences in body size, and therefore, *T*_b_s were pooled together for each population (Table 1). Analysis of variance among populations *T*_b_s revealed significant differences (*F*_9,463_ = 6.421, *p* < 0.001; Figure 2). In particular, we found that the populations from Atsitsa (main island of Skyros) and Atsitsa islet achieved the lowest body temperatures in the field (30.24 ± 3.72 °C and 30.39 ± 1.85 °C, respectively) whereas, Palamari and Molos the highest (33.20 ± 2.69 and 33.06 ± 2.48 °C, respectively; Games Howell test). The overall pattern indicated higher *T*_b_ divergence on the mainland (ranging from 30.24 to 33.20 °C) compared to islet (30.39 to 32.78 °C) lizard populations (Bartlett test of variances; *K*^2^ = 5.40, *df* = 1 and *p* < 0.05) (Table 1).

Mean *T*_e_, as expected, was significantly higher in dunes (i.e., Palamari and Molos biotopes) and lower in the pine forest (i.e., the site of Atsitsa) (permANOVA; based on 9999 iterations, *p* < 0.001; Table 1). The observed differences in *T*_e_ values between sites reflected the differences in habitats, vegetation type, and/or coverage. To take into account the habitat’s thermal heterogeneity, we further analyzed the *T*_e_-records based on the nine solar and substrate combinations on which the models were placed. Pairwise comparisons revealed that only sunlight exposure but not substrate type significantly affected *T*_e_s (permANOVA, all *p* < 0.05) in all locations.

### 3.2. Lab Measurements (T_pref_ and T_set_)

Preferred body (*T*_p_) and set-point (*T*_set_) temperatures showed no differences between sexes for all sites (all *P*s > 0.05), and so the data for each population were pooled together. Permutation ANOVA with 9999 iterations showed that *T*_pref_ (*p* < 0.001) and *T*_set_ (*p* < 0.001) differed significantly across populations with islet lizards achieving in general higher *T*_pref_ and *T*_set_ than their mainland peers (fdr correction; Table 1 and Figure 2). When body size was taken into account [28] as a covariate, the differences remained (*T*_pref_: *F*_9,202_ = 11.325, *p* < 0.001 and *T*_set_: *F*_9,202_ = 8.12, *p* < 0.001). Set-point temperatures received values in a narrower temperature window for islet (31.30 °C to 35.59 °C; *N* = 136) than the mainland (29.75 °C to 35.70 °C; *N* = 124) populations (Bartlett test; *K*^2^ = 39.56, *df* =1 and *p* < 0.001; Table 1).

### 3.3. Effectiveness of Thermoregulation

The mean deviation of *T*_b_ from *T*_set_ (${\bar{d}}_{b}$) showed significant differences across populations (*F*_9,463_ = 5.55, *p* < 0.001; GLM), with mainland ones demonstrating, in general, higher accuracy of thermoregulation than islet kins (Table 1). On islets, the majority of *T*_b_s were lower than the minimum *T*_set_ (on average 56% of the time; 43% to 69%), whereas, on mainland populations, the distribution of *T*_b_s around *T*_set_ was more equally distributed (on average 30% were lower and 21% were higher than the minimum and maximum *T*_set_, respectively).

The mean deviation of *T*_e_ from Tset (${\bar{d}}_{e}$) also differed significantly between populations (*F*_9,20710_ = 368.79, *p* < 0.001; GLM), with mainland ${\bar{d}}_{e}$ values (ranging from 5.07 °C to 8.00 °C) being considerably higher than islet values (ranging from 4.61 °C to 6.88 °C), indicating the poorer thermal quality of the former (Table 1 and Figure 2).

**Figure 3:**
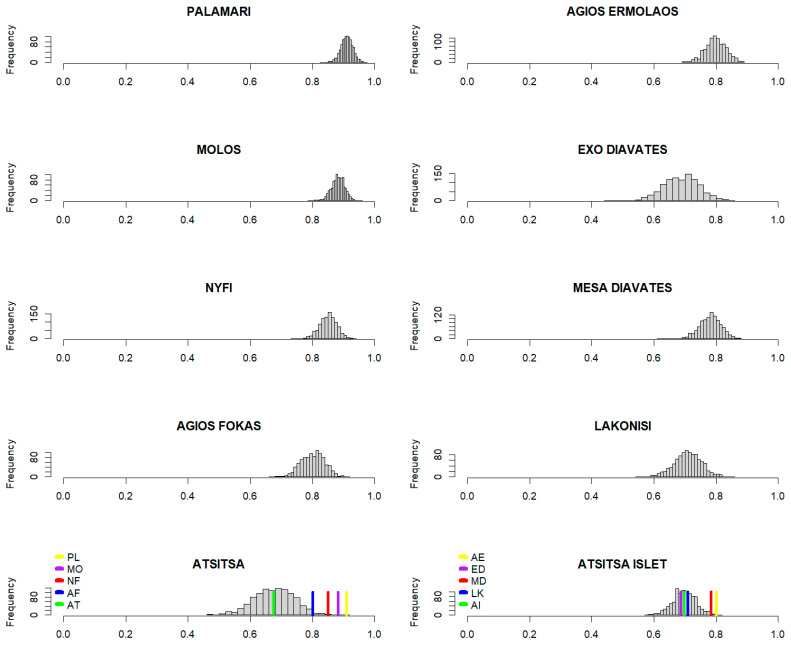
Distribution plot of E bootstrapped values showing the overlaps among populations for islet and mainland sites.

The effectiveness of thermoregulation differed among populations (Table 1 and Figure 3). Mainland lizards, but not the Atsitsa population, exhibited a general pattern of higher *E* values (ranging from 0.80 to 0.91) than islet lizards (ranging from 0.69 to 0.80) (Figure 3). Atsitsa (0.67) lizards achieved the lowest effectiveness of thermoregulation along with Exo Diavates (0.69), Atsitsa Islet (0.70) and Lakonisi (0.71) lizards. Bootstrap resampling correcting for pairwise comparisons for islet populations revealed almost no significant differences across islets besides the lower E values of Exo Diavates (ED) and Atsitsa Islet (AI) from Agios Ermolaos (AE) (973 and 980 E bootstrapped values, respectively, were lower for ED and AI than AE). On the other hand, the effectiveness of thermoregulation among mainland populations demonstrated high variation and significant differences, with Atsitsa population performing the lowest effectiveness of thermoregulation, followed by Agios Fokas and Nyfi lizards, whereas Palamari and Molos demonstrated the highest *E* values (Table 1). The index ${\bar{d}}_{e}$ − ${\bar{d}}_{b}$ [39] corroborated the above findings.

## 4. Discussion

In this study, we aimed to investigate the small island effect of thermoregulatory strategy and thermal preferences in a Mediterranean endemic island lizard. Despite the high thermal homogeneity of islets (*T*_e_s), the quality of their thermal habitat (*d*_e_) was either similar or even higher than that of Skyros environments, contrasting our initial hypothesis. As expected, islet lizards differ in their thermoregulatory accuracy, precision, and effectiveness and select higher temperatures from their mainland kins, a possible adaptive response [18] of their thermal profile to handle islet particularities. The latter advocates the evolutionary lability in the thermal biology of *P. gaigeae*. 

The comparison of habitats’ thermal quality, as described by *T*_e_s, between the islet and “mainland” sites yielded, as predicted, substantial differences, but not in the manner we anticipated. Islets, opposite to our initial prediction, had milder thermal conditions (on average, mean des were lower on islets [19]), with less temperature fluctuations and less extreme values compared to Skyros ‘mainland’ habitats (*d*_e_s ranged from 4.61 to 6.88; Table 1). We think that the reason for this inconsistency should be attributed to the very short distance of the focal islets from Skyros, which, in most cases, is less than a mile. Thus, Skyros, a mountainous island with summits of 800 m, serves as a wind barrier that protects islets from the high winds, controlling high environmental temperatures and daily fluctuations [26,43,44]. By contrast, the thermal profile of Skyros ‘mainland’ sites showed a more inconsistent thermal pattern, with high- and low-quality habitats (*d*_e_s ranged from 5.07 to 8.00). Interestingly, we found that the high and dense vegetation of Atsitsa (pine forest), Agios Fokas (tall maquis and pine trees), and Nyfi (tall maquis with phrygana) sites cool the environment and reduce the heat (Table 1). On the other hand, the rocky grasslands and semi-dunes of Molos and Palamari accumulate heat in the environment, resulting in less favorable thermal habitats (Table 1). Together, these differences could explain the observed *d*_e_ variation that was recorded between Skyros habitats.

To better understand the observed differences in habitat thermal quality across the Skyros Archipelago, one should also take into account the distribution and fluctuations of operative temperatures [19]. In our study, *T*_e_s fluctuations (*T*_e_s) recorded on islets were less extreme than the mainland sites: *T*_e_s readings on Palamari and Molos skewed well above the upper thermal limit and were higher than *T*_set_ 75% of the time, followed by Nyfi and Agios Fokas sites with 62% on average. This lack of extreme *T*_e_ values on islets, coupled with the lower standard deviation of the mean *T*_e_s and the lower inter-population *T*_e_ differences (Table 1), further indicates the higher thermal quality of islets. While islet ecosystems are in general homogenous, with few retreat sites and places available for basking [32], the different habitats on Skyros Island provide a plethora of shelters and basking places, which are located in different elevations and slopes and have strikingly different vegetation structure, coverage, and floristic elements [45]. Thus, Skyros encompasses diverse thermal habitats, a fact explaining the observed *T*_e_ variation.

Body temperatures that *P. gaigeae* achieved in the field showed significant differences across the landscape and were in accordance with previous reports on the focal species [28] and within the *T*_b_ range of other *Podarcis* [26,32,44,46]. In particular, we found that lizards from the semi-dune and less grass-shaded habitats of Palamari and Molos achieved the highest *T*_b_s (33.19 °C and 33.05 °C, respectively). By contrast, Atsitsa (on Skyros Island, a pine forest with very dense tree canopy and more than 70% vegetation coverage that provides extensive well-shaded thermal (micro)habitat(s)) and Atsitsa islet (very dense vegetation of phrygana) lizards that are exposed to shadier and cooler habitats (*T*_e_s are 34.92 °C and 37. 66 °C, respectively) had the lowest *T*_b_s (30.24 °C and 30.39 °C, respectively). Especially for Atsitsa lizards, only 26% of their *T*_b_s were within the *T*_pref_ interquartile range (i.e., *T*_set_), whereas for the rest of the populations, this value ranged from 38% to 49%. Noteworthy, the Atsitsa islet hosts a dense rabbit population that could also challenge lizards from reaching their preferred temperature. Lastly, lizards that occupy more favorable ecosystems in terms of structure, vegetation, and ambient temperatures (i.e., Nyfi, Agios Fokas, Agios Ermolaos, Mesa/Exo Diavates, and Lakonisi) maintained their body temperatures closer to their optimal values. 

In accordance with Sagonas et al. [28], our results indicate effective thermoregulation for all *P. gaigeae* populations (all *E*s > 0.6). When animals are unable to thermoregulate, they select microhabitats randomly, and *E* approaches zero, whereas a value close to one indicates animals that actively select appropriate microhabitats and that successfully regulate their body temperature [19]. Both indices of thermoregulation yielded similar results, with the “mainland” Atsitsa population achieving the lowest *E* value, followed by the islet populations (ranging from 0.69 to 0.80), while Agios Fokas, Nyfi, Palamari, and Molos reached the highest *E*s (ranging from 0.80 to 0.91) (Table 1 and Figure 3). However, the approach proposed by Blouin-Demers and Weatherhead [39] suggested less effective thermoregulation for islet lizards as the respective values of mainland populations, especially those from Nyfi, Palamari, and Molos, were 1.5 to 2 times higher over those of the islets and “mainland” Atsitsa. Overall, our findings on the effectiveness of thermoregulation oppose our second hypothesis, as islanders demonstrated lower *E*s compared to their mainland kin. 

Lizards are known to thermoregulate more accurately in thermally heterogeneous places where they can move across a spectrum of microhabitats to maintain *T*_b_ close to their *T*_pref_ [47,48]. In contrast, thermally challenging environments with few high-quality thermal spots and shelters often impose shifts in lizards’ thermal optima and thermoregulatory strategies to cope with the habitat’s low thermal quality [32,44,49]. In agreement with this, islet *P. gaigeae* have shifted their thermal preferences, buffering the impact of the harsher but more homogenous environment in terms of temperature and habitat. As such, islet lizards can maintain their *T*_b_s within or close to *T*_set_ without devoting too much effort (i.e., low E values) compared to their mainland conspecifics (Table 1). Resource availability [17], predation pressure [50], and intraspecific competition [28] are also known to affect the regulation of ectotherms’ body temperature and their effectiveness of thermoregulation as they influence the time lizards dedicate to reach their *T*_set_. Contrary to islet populations where predation is minimal [51], Skyros hosts numerous lizard-eating predators, including snakes, birds, and domestic cats [31]. As such, the pressure of ‘mainland’ *P. gaigeae* lizards to keep performance at its peak by thermoregulating in high effectiveness may be of particular importance for their survival. 

Nevertheless, the effectiveness of thermoregulation showed significant differences between Skyros populations (Table 1; *E* values ranged from 0.67 to 0.91). Wilms et al. [52] suggested that lizards thermoregulate more effectively when the thermal quality of the habitat is low. Likewise, Adamopoulou and Valakos [53] proposed that the structurally poor environment of sand dunes forces *P. milensis* (the sister species of *P. gaigeae*) to thermoregulate with great success (*E* = 0.95) in order to avoid overheating. According to these studies, Molos and Palamari lizards thermoregulate more effectively and accurately compared to Nyfi and Agios Fokas, which inhabit more favorable environments (Table 1), while Atsitsa lizards displayed the lowest effectiveness. 

The fact that the different populations of *P. gaigeae* are active at different body temperatures and exploit varied thermal habitats could explain the observed shifts in *T*_pref_ (Table 1). Preferred body temperatures have pronounced effects on the estimation of all thermoregulation indices (*d*_e_, *d*_b_, and *E*) and define the ability of lizards to thermoregulate accurately and effectively [19]. While the idea that evolutionary constraints play an important role in saurian thermal biology is gaining ground [44,54,55,56,57,58,59,60], as the evolution of thermal biology often requires adjustments in the performance of numerous physiological, biological, and life-history traits [61,62,63,64], our data suggest that the thermal physiology of *P. gaigeae* is evolutionarily flexible (see also [28]). In other words, our findings comply with the ‘labile’ view, according to which the thermal physiology is plastic and responds to directional selection [57,65]. The latter also supports the ability of *P. gaigeae* to colonize a wide array of thermal environments. Surprisingly, we found little or no effect of body size on *P. gaigeae* thermal preferences (to satisfy the increased metabolic needs of a larger body, lizards are often expected to select for higher body and preferred temperatures [62,66]). Marquet et al. [67] found that Andean *Liolaemus* lizards exposed to similar thermal environments thermoregulate in a similar way despite their different body sizes. 

Our findings, taken together with those of other studies [26,32,44,49,53], highlight the importance of habitat structure and quality on lizards’ thermoregulatory behavior. Using multiple islet and “mainland” lizard populations from diverse habitats across the Skyros Archipelagos, we found that *P. gaigeae* can directionally respond to changes in the thermal environment. This is particularly important not only to understand species’ spatial distribution but also to better predict how they will respond to the current ongoing climate change that causes alterations in ectotherms’ thermal environment. Given its restricted distribution and isolation, the species could be considered vulnerable to such changes. Future research should now focus on unraveling whether the observed shifts in thermal preferences in *P. gaigeae* are adaptive plastic responses [18] to thermal habitat-mediated selection to handle islet particularities.

## 5. Conclusions

We found that the thermal preferences of *P. gaigeae* tend to differ across different habitats. These results support the labile view on the evolution of thermal physiology [57] for this species, according to which *T*_set_ is not stable and depends on the particular conditions of the habitat. It also appears that thanks to the more relaxed predation pressure and higher thermal quality, islet lizards thermoregulate with less accuracy and effectiveness, which may allow them to spend more time foraging [68,69]. “Mainland” Skyros lizards, on the other hand, living in a more challenged and complex environment, thermoregulate with higher accuracy because the selection for an effective thermoregulation is strong.

## Data Availability

The data presented in this study are available on request from the corresponding author.
